# Basophils may as a risk factor for upper gastrointestinal cancer: a Mendelian randomization (MR) study

**DOI:** 10.3332/ecancer.2024.1799

**Published:** 2024-11-14

**Authors:** Pengkhun Nov, Wandan Li, Duanyu Wang, Socheat Touch, Samnang Kouy, Peizan Ni, Qianzi Kou, Ying Li, Chongyang Zheng, Arzoo Prasai, Wen Fu, Kunpeng Du, Syphanna Sou, Jiqiang Li

**Affiliations:** 1 Department of Radiation Oncology, Oncology Center, Zhujiang Hospital of Southern Medical University, No 253 Mid Gongye Ave, Haizhu District, Guangzhou 510282, Guangdong Province, China; 2 Department of Radiation Oncology, Luang Mè Hospital of University of Health Sciences, Street 109, Phnom Penh 120110, Cambodia; a https://orcid.org/0000-0002-0684-7291; b https://orcid.org/0000-0002-585-5911; † These authors contributed equally to this work

**Keywords:** esophageal cancer, gastric cancer, peripheral circulating blood cells, Mendelian randomization (MR), genome wide association study (GWAS)

## Abstract

**Objective:**

Upper gastrointestinal (UGI) cancers, including esophageal (EC) and gastric (GC) cancers, pose a significant global health challenge. Previous studies have indicated a fundamental correlation between basophil count and the risk of UGI cancer. However, confirming a causal relationship demands further investigation. Mendelian randomization (MR) provides a critical method for evaluating the possible causal connections between peripheral circulating blood cells (PCBCs) and UGI cancer.

**Method:**

Our study comprehensively employed a two-sample MR analysis. We used publicly available genetic data to survey the causal association between PCBC and UGI cancer. We used inverse variance weighting and weighted median for MR analyses and sensitivity analyses to assess heterogeneity and pleiotropy.

**Results:**

In terms of the association between PCBCs and UGI cancer, we found that basophils count (EC: OR = 1.416, 95% CI = 1.125−1.783, *p* = 0.003; GC: OR = 1.623, 95% CI = 1.052−2.505, *p* = 0.029) were all strongly correlated with both EC and GC. Interestingly, Basophil count was a risk factor for both EC and GC. However, no significant correlations were seen between eosinophil, monocyte, lymphocyte or white blood cell count and UGI cancer.

**Conclusion:**

The findings of this research corroborate the idea that basophils might serve as a fundamental risk factor for UGI cancer. Further exploration of the underlying mechanisms driving this relationship could provide crucial understanding helpful in creating prospective preventive and treatment methods for UGI cancer.

## Introduction

Upper gastrointestinal (UGI) cancers, particularly esophageal cancer (EC; 510,716 new cases in 2022) and gastric cancer (GC; approximately 1 million new cases in 2022), present substantial global health challenges, marked by significant morbidity and mortality rates [1]. Identifying novel risk factors and underlying mechanisms is crucial for developing effective prevention and early detection strategies [2, 3]. While numerous studies have explored the correlation between various types of peripheral circulating blood cells (PCBCs) and cancer risk, the causal nature of these relationships remains under active investigation [4, 5].

Basophils, a type of granulocyte involved in immune responses and allergic reactions, have been highlighted in recent research as potentially linked to an increase risk of UGI cancer [6]. Previous observational studies have suggested that a higher basophil count is related to a higher risk of UGI cancer [7]. However, these observational studies are susceptible to deviation and confounders, thus limiting their ability to confirm causality [6, 7]. Mendelian randomization (MR), a method that provides conclusive causal evidence by analysing the association of genetic variation with exposure and discovery, offers a promising replacement for randomised controlled trials (RCTs) [8]. By utilising genetic variants that affect basophil levels, MR facilitates a more rigorous examination of basophils’ role in UGI cancer development, minimising biases commonly present in observational studies [9–11].

In the present study, we investigated the histophysiological and pathophysiological engagement of PCBCs in the carcinogenesis of cancers of the GI tract using MR analysis, which was achieved through a recent Genome-Wide Association Study (GWAS) focusing on PCBCs and UGI cancers and statistical summaries from the UK Biobank [12]. Our study is dedicated to exploring the causal association between PCBCs and UGI cancers, with a special focus on those in tumor initiation, progression and treatment resistance. We present an extensive MR study that not only identifies specific PCBCs associated with UGI cancers but also addresses the constraints in current research. Our goal is to provide valuable insights that could refine future methodologies and advance etiological research. This work is intended to support precision prevention, control and the development of innovative therapeutic approaches. Additionally, we aim to develop personalised treatment strategies targeting the specific PCBCs vulnerabilities of UGI cancer.

## Materials and methods

### Study design

**Figure 1.**
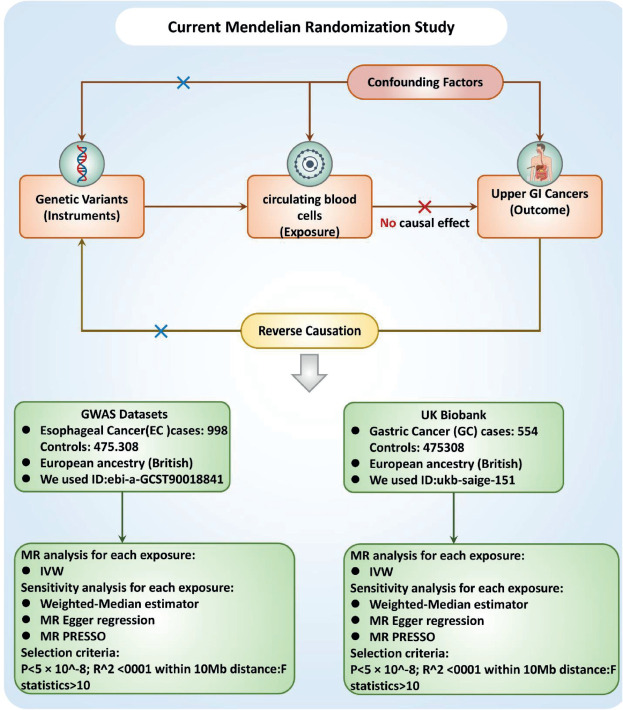
Study design flowchart. The first assumption is that the instrument variables are strongly related to the exposure. The second assumption specifies that the instrument variables are not associated with any confounders. The third assumption establishes that the instrument variables influence the outcome solely through the exposure. Abbreviations include SNPs for single-nucleotide polymorphisms, LD for linkage disequilibrium, IVW for inverse variance weighted and weighted median, MR-Egger and MR-PRESSO.

Causal associations between PCBC and UGI cancer were assessed using a two-sample MR analysis. MR uses genetic variants as a proxy for risk factors. To ensure reliable causal inferences, the instrumental variables (IVs) used in MR must meet three key assumptions: (1) the genetic variant must be directly related to the exposure; (2) the genetic variant is not related to potential confounders between the exposure and the finding; and (3) the genetic variant affects the finding only through the exposure and not through other pathways Figure 1.

### Data sources for exposure and outcome

GWAS statistical summary for each PCBCs trait is publicly available from the GWAS catalogue (accession numbers: GCST0001391 to GCST0002121) [12]. We used keywords for each cancer to find IDs from (https://gwas.mrcieu.ac.uk/). Cancer IDs included: ebi-a-GCST90018841 (EC), ukb-saige-151 (GC). Finally, we used summary statistics from the latest large-scale Blood Cell Characterisation Genome Study (BCX), conducted by the blood cell consortium with participants of European ancestry [12]. The GWAS database is a fully comprehensible gathering of genetic variants and their connections to a diversity of traits or diseases. It provides a powerful resource for researchers and clinicians who are interested in developing an understanding of the genetic basis of complex traits and diseases. From this GWAS, we derived genetic variants associated with circulating leukocyte, lymphocyte, monocyte, neutrophil, eosinophil and basophil count levels. Based on EC IDs, we downloaded EC data for 476,306 Europeans (*n* = 998 case patients and 475,308 control participants) from the GWAS (https://www.ebi.ac.uk/gwas/). The UK Biobank is a large-scale biomedical database and research resource that is designed to allow researchers to seeking the hereditary, environmental and lifestyle factors that influence various diseases and health outcomes. The database includes health and genetic information from over 500,000 participants in the United Kingdom, making it one of the most comprehensive biomedical resources of its kind. Based on the ID of GC, we downloaded the data from UK Biobank including 393,926 European individuals (*n* = 554 case patients and 393,372 control participants) for EC (https://www.ukbiobank.ac.uk).

### Instrument selection

We implemented a more stringent correlation threshold (*p* < 5 × 10^-9) for the selection of genetic IVs, given the large number of single nucleotide polymorphisms (SNPs) that have genome-wide significance (*p* < 5 × 10^-8) for PCBCs traits [12]. These IVs were identified by grouping them according to the 1,000 Genomes Project’s Linkage Disequilibrium (LD) reference panel, with a threshold of *R*^2 < 0.001 at a distance of 1,000 kilobases (kb). Given the relatively limited size of the GWAS data from PCBCs, we employed a *p* value threshold of 5 × 10^ -8 *p*-value threshold and a less significant clustering threshold (*R*^2^ < 0.001 at a distance of 1,000 kb) [13]. To ensure the reliability of the tool, we selected IVs with an* F*-statistic of more than 10, identifying them as strong tools for subsequent analyses. We then extracted these IVs from the summary statistics regarding UGI cancer outcomes according to the methodology of previous studies [14], excluding any IVs with a potential pleiotropic effect (*p* < 10^-5) on UGI cancer. To maintain consistency in the analyses, we synchronised the SNPs in the exposure dataset and in the findings dataset to ensure that the effect estimates of the same effect allele remained consistent. Alleles with intermediate effect frequencies (EAFs >0.42) or SNPs incompatible with the allele were excluded from our analyses [13].

### Statistical analysis

In our research, we used a series of Genetic Variants as IVs rather than relying solely on an allele score. This approach was chosen to thoroughly examine key assumptions, uncover potential pleiotropy and facilitate more effective sensitivity and multivariable MR analyses [11]. To assess the consistency of our findings under different assumptions about heterogeneity and pleiotropy, we utilised four distinct MR methodologies: The inverse variance weighted (IVW), Weighted Median, MR-Egger and MR Pleiotropy Residual Sum and Outlier (MR-PRESSO). The IVW method, employing a random-effects model, served as the primary analysis framework for all four sets of IVs. Cochran’s *Q* statistical measurements and the appropriate *p*-values were used to test for heterogeneity among the elected IVs.

Our study also included analyses with more stringent conditions. In the IVW approach, under the hypothesis that all genetic variants are effective, if many SNPs are influenced by horizontal pleiotropy, bias will occur [15]. Conversely, the weighted median approach, effective when less than 50% of variants exhibit horizontal pleiotropy, presumes most genetic variants are valid [16]. In cases where over 50% of variants are affected by horizontal pleiotropy, we evaluated the strength of our genetic instruments through *F* statistics, considering a mean *F* < 10 indicative of weak IVs [17].

Furthermore, to rule out the effect of horizontal multidimensionality, we used a frequently utilised approach (MR-Egger), which implores the presence of horizontal multidimensionality if the interception term is statistically significant [18]. We also used the powerful (MR-PRESSO method to eliminate potential horizontal pleiotropic outliers, which could have affected the estimation results of the MR-PRESSO package [19]. Additionally, Steiger-filtering analyses were conducted to detect and eliminate genetic variants more strongly associated with the outcome than the exposure, demonstrating possible reverse causality [20].

All statistical analyses were conducted with R software 4.3.1 (R Foundation) and specific R packages (‘TwoSampleMR’ and ‘MR’) tailored for MR analysis [21, 22].

## Results

### The causal role of PCBCs and UGI cancers

#### The causal role of PCBCs and EC

**Figure 2.**
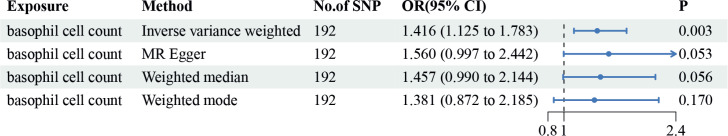
The causal estimation between basophil cell count and EC. We selected IVW as a primary method *p* < 0.05 showed statistical significant; OR value >1 indicated a risk factor.

Figure 2 summarises the causal effect estimates of circulating blood count on susceptibility to EC. We found a strong positive causal association between EC risk circulating basophil cell count (OR = 1.416, 95% CI = 1.125–1.783, *p* = 0.003). However, no significant correlations were observed between monocyte, lymphocyte, eosinophil or neutrophil counts and EC susceptibility.

**Figure 3.**
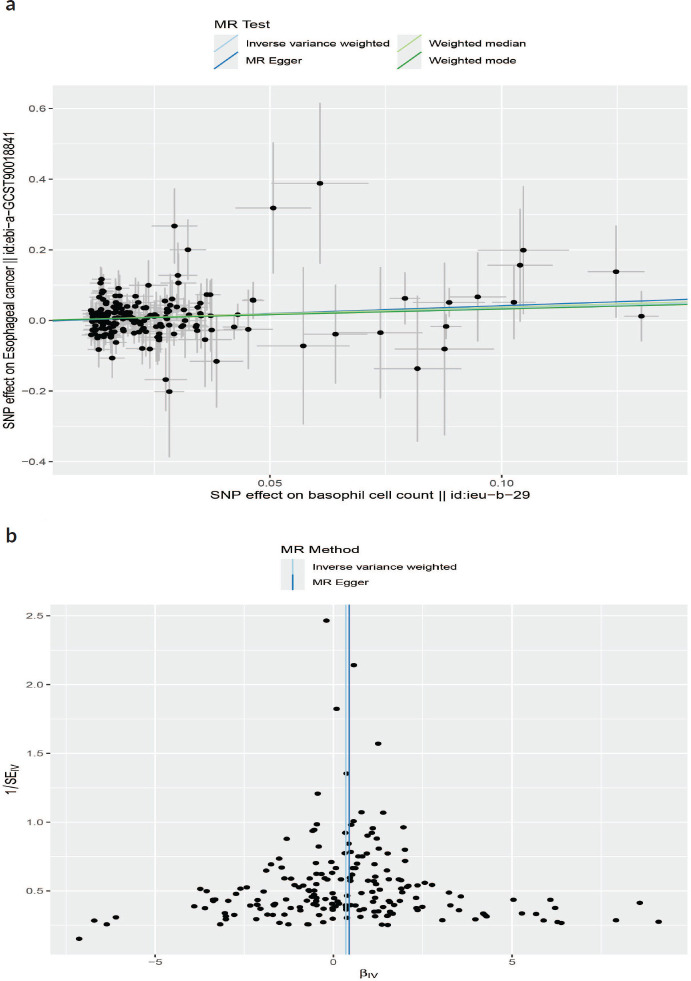
(a): The scatter plot demonstrating the genetic associations of basophil cell count on the risk of EC. (b): The funnel plot represents IVs for each significant causal relation between basophil cell count and EC.

Neither the MR-Egger intercept test nor Cochran’s *Q* test showed pleiotropy or heterogeneity (Tables S1 and S2). Additionally, scatter plots Figure 3A and funnel plots Figure 3B were employed to analyse the data, effectively reducing the likelihood of potential outliers and horizontal pleiotropy affecting any of the identified basophil cells.

## The causal role of PCBCs and GC

**Figure 4.**
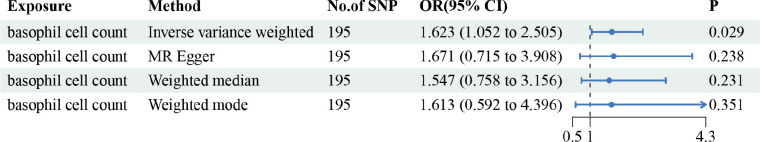
The causal estimation between basophil cell count and GC. We selected IVW as a primary method *p* < 0.05 showed statistically significant; OR value >1 indicated a risk factor.

**Figure 5.**
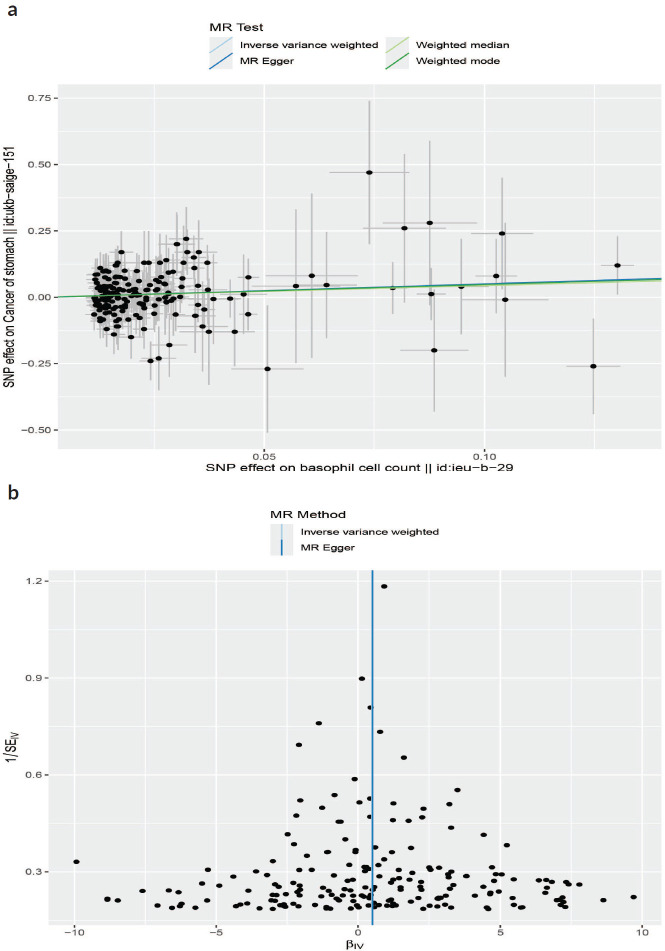
(a): The scatter plot demonstrating the genetic associations of basophil cell count on the risk of GC. (b): The funnel plot represents IVs for each significant causal relation between basophil cell count and GC.

IVW results suggested a risk effect of basophil cell count (OR = 1.623, 95% CI = 1.052–2.505, *p* = 0.029) on GC risk (Figure 4). However, no significant correlations were seen between basophil cell count, monocyte cell count, lymphocyte cell count or neutrophil cell count and GC. Neither the MR-Egger intercept test nor Cochran’s *Q* test revealed pleiotropy or heterogeneity (Tables S3 and S4). Additionally, scatter plots Figure 5A and funnel plots Figure 5B were employed to analyse the data, effectively reducing the likelihood of potential outliers and horizontal pleiotropy affecting any of the identified basophil cells.

## Discussion

MR analysis has been frequently employed to illustrate possible causality between risk factors and diseases [19]. In this MR study, we used MR to investigate the causal correlations between PCBCs and UGI cancer. The results indicate that higher basophil levels may elevate the risk of UGI cancer, thus expanding our comprehension of the potential cancer-causing roles of these cells. Basophils, a type of white blood cell, are naturally present in small quantities in the bloodstream. They are essential for the immune system and play a crucial role in both hypersensitivity reactions and fighting against parasitic infections. Basophils are recognised for their ability to produce and release inflammatory substances like histamine and cytokines, which contribute to the body’s immune response [23, 24]. Elevated and reduced levels of basophils in peripheral blood may be linked to the progression of specific types of solid tumors in humans [25, 26].

Basophils derived from stem progenitor cells are found in the bone marrow [27–29]. In both humans and mice, interleukin-3 (IL-3) serves as the primary growth factor essential for basophils maturation [30–33]. Several studies have indicated that basophils in these species can be cultured *in vitro*. This is achieved by incubating bone marrow cells or, in humans, CD34* precursors with IL-3 for a duration of 10–14 days [30, 34–36]. Although IL-3 plays a crucial role in the establishment of basophils from their precursors, additional growth factors contribute to their expansion and function. For instance, the combination of Feline McDonough Sarcoma-like tyrosine kinase 3 ligand with IL-3 has been demonstrated to enhance basophil production in cultures [37]. Moreover, research by Siracusa *et al* [38] revealed that mouse basophils could be produced using thymic stromal lymphopoietin (TSLP), which involves the heterodimeric TSLP receptor (TSLPR/IL-7Ro) [38]. This study further suggests that IL-3 and TSLP can stimulate the polarization of two different types of basophils in mice, each characterised by unique gene expression profiles and functions [39].

In terms of the function of PCBCs in UGI cancer, basophils have not generated much interest due to their scarce dissemination and relatively unexplored functions. Basophils perform an essential role in hypersensitivity diseases and type 1 hypersensitivity responses [40]. In addition, they are recruited to tissues following parasitic, bacterial and viral infections. Subsequent establishment of basophil removal antibodies and basophil-deficient mouse models has recently provided a greater understanding of basophil biology beyond allergic reactions [41]. Consequently, a potential relationship between basophils and tumor immunity may also exist [42]. Basophils not only enhance humoral immune function but also liberate intracellular agents causing anti-tumor immunity [43–45], including the chemokines CCL3 and CCL4, which expedite CD8+ T-cell infiltration [43], and the chemokines TNF-α and IL-6, which enhance inflammatory anti-tumor responses [46, 47]. Some studies have reported that basophils have favorable survival outcomes in melanoma [48, 49], ovarian cancer [50], endometrial [51], sarcoma cancers [52], non-small cell lung cancer [53] and glioblastoma [54], while basopenia is correlated with a worse prognosis for colorectal cancer [55, 56]. On the other hand, basophilia has been linked to better outcomes in patients with melanoma who undergoing immunotherapy treatment [49]. The above findings are inconsistent with our results demonstrating that basophils were strongly associated with UGI cancer and that increased basophil counts may contribute to reduced survival.

Dvorak’s [57] seminal discovery of piecemeal degranulation in basophils in human pancreatic cancer (PC) set the stage for subsequent investigations. Extensive research has been conducted to explore basophils’ involvement in human pancreatic ductal adenocarcinoma (PDAC) [58]. In those patients with PDAC, basophils expressing IL4 have been found in tumor-draining lymph nodes (TDLNs), and their presence emerged as an independent negative prognosis indicator for patient survival. Investigating basophils’ function in PC was further conducted in basophil-deficient Mcpt8-Cre mice [59] and wild-type (WT) mice. Following PC implantation, cancer was observed in 80% of WT mice but was notably absent in the Mcpt8-Cre mice. The basophils in TDLNs were observed, and the role of cancer-associated fibroblasts in releasing TSLP was noted, which in turn activated dendritic cells (DCs) to generate IL-3 from CD4* T cells. Both DCs and CD14* monocytes secreted CCL7, triggering the migration of basophils into TDLNs. Once activated by IL-3, the basophils assumed a pro-tumorigenic role, secreting IL-4 and thereby promoting Th2 and M2 polarization. In another interesting study, in the advanced stage of chronic myeloid leukemia (CML), basophilia was also found to be accumulated [60] and a notable reduction in the IKAROS, a transcription factor in these patient’s bone marrow, was observed [61]. CML patient-derived basophils were found to express HIGF, promoting the expansion of CML cells [62]. Furthermore, in a mouse CML model, basophil-derived CCL3 was implicated in promoting CML progression [63]. Basophilia was recognised as an independent predictor of acute myeloid leukemia that progressed from myelodysplastic syndrome [64, 65]. These observations are supported by our results that basophils are the risk factors in UGI cancer, where elevated basophil counts may correlate with diminished survival.

In other solid tumor types, such as GCs [7] and prostate [4], adverse effects related to tissue infiltrating or circulating basophils have been reported. Notably, in bladder cancer patients, the initial basophil count has been identified as a predictor of recurrence following tumor resection and bacillus Calmette–Guérin therapy [5]. In contrast, a study using a mouse model of breast cancer (BC) revealed that basopenia, or a low basophil count, correlated with an increased number of pulmonary metastases [66]. However, no significant correlation has been established between basophils and prognosis in BC patients [67]. This variation in findings suggests that the local tumor microenvironment might dictate whether basophils exhibit pro-tumor or anti-tumor effects, potentially explaining their inconsistent impact on survival across different cancer types [26]. Basophils play a crucial role in supporting humoral immunity, mainly by secreting molecules that modulate B-cell function. Activated basophils can express CD40L, IL-4 and IL-6, which are vital in sustaining B-cell proliferation and boosting IgM and IgG1 production. Research by Rodriguez Gomez *et al* [45] have demonstrated that basophils, both *in vitro* and *in vivo*, are instrumental in promoting plasma cell survival [24, 45]. Our research aligns with these findings, particularly highlighting a strong association of basophils with UGI cancer, supporting the broader implications of basophils in cancer dynamics.

## Strength and limitations

Our two-sample MR analysis was designed to analyse the causal relationships between PCBCs in UGI cancer using large-sample GWAS and UK Biobank data. This design reduced the limitations of traditional observational studies by eliminating the influence of confounding factors and reverse causality. In addition, MR mitigates the representativeness and feasibility issues inherent to RCTs. However, there are several limitations to this study. First, it depended on publicly accessible GWAS and UK Biobank data, which prevented further exploration of the impact of other relevant factors on UGI cancers, such as gender, age and body mass index [68]. Second, these findings can only be generalised to European populations, as that was the sample present in the original GWAS and UK Biobank, and further study is needed in different ethnic subgroups [69]. Third, even with multi-sensitivity analysis, horizontal pleiotropy could not be comprehensively evaluated. Finally, we employed wider thresholds for assessing the findings, which may grow false positives but also enable a more critical assessment of the association between circulating blood cell characteristics and UGI cancer. In addition, this study covered a wide range of circulating blood cells, but the functions and mechanisms of circulating blood cells in disease are not yet fully understood, which limits our interpretation of the findings from the MR analysis.

## Conclusion

In summary, our MR analysis offers evidence suggesting a potential causal relationship between PCBCs and the risk of UGI cancer. The results highlight the possible role of basophils as a risk factor for UGI cancer, emphasising the importance of further research to elucidate the underlying mechanisms. Understanding the causal link between basophils and UGI cancer may offer valuable insights for the development of targeted preventive and therapeutic strategies, potentially contributing to improved outcomes for individuals at risk of this disease.

## Conflicts of interest

No competing interests.

## Ethics approval

Not applicable because the present study extracted data from database.

## Author contributions

1. Pengkhun Nov design, analysis and interpretation of data.
2. Wandan Li revise and agree to publish.
3. Duanyu Wang drafting of the paper.
4. Syphanna Sou revise and agree to publish.
5. Socheat Touch revise and agree to publish.
6. Samnang Kouy revise and agree to publish.
7. Qianzi Kou revise and agree to publish.
8. Ying Li revise and agree to publish.
9. Kunpeng Du revise and agree to publish.
10. Jiqiang Li revise and agree to publish.

## Data availability

All the data for this paper is available in the GWAS and UK biobank database.

## Patients consent for publication

Not available because we used public database analyse this study.

## Supplementary tables

**Table S1.** The pleiotropy of causal relationship between basophil and EC. The *p*-values for the MR-Egger intercept were above 0.05, suggesting that no significant pleiotropy effects were found.

Exposure	Egger_intercept	SE	pval
basophil cell count || id:ieu-b-29	−0.001505142	0.006200437	0.808548701
monocyte cell count || id:ieu-b-31	−0.001403486	0.003515935	0.690005147
lymphocyte cell count || id:ieu-b-32	0.000821016	0.004785895	0.863892238
eosinophil cell count || id:ieu-b-33	−0.005860566	0.004378537	0.181700556
neutrophil cell count || id:ieu-b-34	−0.006988986	0.004891447	0.154147906

**Table S2.** The heterogeneity of causal relationship between basophil and EC. The *p*-values for the Cochran’s Q yielded were above 0.05, suggesting that no significant heterogeneity effects were found.

Exposure	Method	*Q*	Qdf	Q_pval
basophil cell count || id:ieu-b-29	MR Egger	141.0684498	143	0.529994006
basophil cell count || id:ieu-b-29	IVW	141.1273762	144	0.552131124
white blood cell count || id:ieu-b-30	MR Egger	348.5353123	338	0.334817873
white blood cell count || id:ieu-b-30	IVW	353.080672	339	0.288089873
monocyte cell count || id:ieu-b-31	MR Egger	336.8186378	350	0.684145589
monocyte cell count || id:ieu-b-31	IVW	336.9779813	351	0.695392153
lymphocyte cell count || id:ieu-b-32	MR Egger	407.529816	346	0.012605061
lymphocyte cell count || id:ieu-b-32	IVW	407.5644785	347	0.013819915
eosinophil cell count || id:ieu-b-33	MR Egger	297.5814706	317	0.776649093
eosinophil cell count || id:ieu-b-33	IVW	299.372988	318	0.76637903
neutrophil cell count || id:ieu-b-34	MR Egger	311.3277161	286	0.145268396
neutrophil cell count || id:ieu-b-34	IVW	313.5500296	287	0.134935357

**Table S3.** The pleiotropy of causal relationship between basophil and GC. The *p*-values for the MR-Egger intercept were above 0.05, suggesting that no significant pleiotropy effects were found.

Exposure	Egger_intercept	SE	pval
basophil cell count	−0.0007975280	0.010195	0.937726
white blood cell count	−0.0079861412	0.006827	0.24264
monocyte cell count	−0.0011968404	0.005511	0.828149
lymphocyte cell count	0.0089755673	0.006665	0.178682
eosinophil cell count	0.0005035087	0.006925	0.942069
neutrophil cell count	−0.0070981274	0.007169	0.322676

**Table S4.** The heterogeneity of causal relationship between basophil and GC. The *p*-values for the Cochran’s Q yielded were above 0.05, suggesting that no significant heterogeneity effects were found.

Exposure	Method	*Q*	Qdf	Q_pval
basophil cell count || id:ieu-b-29	MR Egger	172.0774212	193	0.858093
basophil cell count || id:ieu-b-29	IVW	172.083541	194	0.869356
white blood cell count || id:ieu-b-30	MR Egger	443.5311811	479	0.875717
white blood cell count || id:ieu-b-30	IVW	444.8997452	480	0.872869
monocyte cell count || id:ieu-b-31	MR Egger	485.7211764	491	0.558736
monocyte cell count || id:ieu-b-31	IVW	485.7683485	492	0.570727
lymphocyte cell count || id:ieu-b-32	MR Egger	441.321151	493	0.95407
lymphocyte cell count || id:ieu-b-32	IVW	443.1348629	494	0.951136
eosinophil cell count || id:ieu-b-33	MR Egger	375.737826	433	0.9781
eosinophil cell count || id:ieu-b-33	IVW	375.743113	434	0.979847
neutrophil cell count || id:ieu-b-34	MR Egger	413.3696256	412	0.471746
neutrophil cell count || id:ieu-b-34	IVW	414.3533065	413	0.472006
